# Accelerated high-dose radiotherapy alone or combined with either concomitant or sequential chemotherapy; treatments of choice in patients with Non-Small Cell Lung Cancer

**DOI:** 10.1186/1748-717X-2-27

**Published:** 2007-07-23

**Authors:** Apollonia LJ Uitterhoeve, Mia GJ Koolen, Rob M van Os, Kees Koedooder, Marlou van de Kar, Bradley R Pieters, Caro CE Koning

**Affiliations:** 1 Department of Radiation Oncology, Academic Medical Center/University of Amsterdam, Amsterdam, The Netherlands; 2 Department of Pulmonary Disease, Academic Medical Center/University of Amsterdam, Amsterdam, The Netherlands

## Abstract

**Background:**

Results of high-dose chemo-radiotherapy (CRT), using the treatment schedules of EORTC study 08972/22973 or radiotherapy (RT) alone were analyzed among all patients (pts) with Non Small Cell Lung Cancer (NSCLC) treated with curative intent in our department from 1995–2004.

**Material:**

Included are 131 pts with medically inoperable or with irresectable NSCLC (TNM stage I:15 pts, IIB:15 pts, IIIA:57 pts, IIIB:43 pts, X:1 pt).

**Treatment:**

Group I: Concomitant CRT: 66 Gy/2.75 Gy/24 fractions (fx)/33 days combined with daily administration of cisplatin 6 mg/m^2^: 56 pts (standard).

Group II: Sequential CRT: two courses of a 21-day schedule of chemotherapy (gemcitabin 1250 mg/m^2 ^d1, cisplatin 75 mg/m2 d2) followed by 66 Gy/2.75 Gy/24 fx/33 days without daily cisplatin: 26 pts.

Group III: RT: 66 Gy/2.75 Gy/24 fx/33 days or 60 Gy/3 Gy/20 fx/26 days: 49 pts.

**Results:**

The 1, 2, and 5 year actuarial overall survival (OS) were 46%, 24%, and 15%, respectively.

At multivariate analysis the only factor with a significantly positive influence on OS was treatment with chemo-radiation (P = 0.024) (1-, 2-, and 5-yr OS 56%, 30% and 22% respectively). The incidence of local recurrence was 36%, the incidence of distant metastases 46%.

Late complications grade 3 were seen in 21 pts and grade 4 in 4 patients. One patient had a lethal complication (oesophageal). For 32 patients insufficient data were available to assess late complications.

**Conclusion:**

In this study we were able to reproduce the results of EORTC trial 08972/22973 in a non-selected patient population outside of the setting of a randomised trial. Radiotherapy (66 Gy/24 fx/33 days) combined with either concomitant daily low dose cisplatin or with two neo-adjuvant courses of gemcitabin and cisplatin are effective treatments for patients with locally advanced Non-Small Cell Lung Cancer. The concomitant schedule is also suitable for elderly people with co-morbidity.

## Background

Worldwide and in Europe lung cancer is the most common cause of cancer related-death with an increasing incidence each year. The majority of patients has Non-Small Cell Lung Cancer (NSCLC) and 75% has advanced disease at the time of diagnosis. Since the mean age at diagnosis is around 66 years it is often found in elderly patients with co-morbidity [1-3].

Adaptation of our treatment policy has been guided by the results of several subsequent EORTC studies in which we participated. The results of EORTC 08844 indicated that combination of radiotherapy with daily low-dose cisplatin is superior to radiotherapy alone or combined with weekly cisplatin [4]. One of the criticisms of that study was that the radiotherapy schedule was given as a split course regimen up to 55 Gy.

In our institute a treatment schedule of 60 Gy in 3 Gy fractions during 26 days using a concomitant boost technique was investigated and appeared to be feasible [5]. A subsequent feasibility study showed that the split period, part of the treatment regimen of EORTC-08844, could be left out. This resulted in a radiotherapy schedule of 55 Gy, given in 20 fractions in an overall treatment time (OTT) of 26 days, combined with daily cisplatin (6 mg/m^2^) [6]. In EORTC-08912, the feasibility of increasing the radiotherapy dose up to 66 Gy in 24 fractions of 2.75 Gy using a concomitant boost technique was demonstrated. Every radiotherapy fraction was preceded by administration of cisplatin 6 mg/m^2^[7]. The standard radiotherapy dose in combination therapy evolved thus from a dose of 55 Gy split course in 1995 to 66 Gy continuously in 1997.

Since 1997 we have participated in EORTC-08972/22973. In this trial sequential versus concurrent radio-chemotherapy has been studied [8]. The radiotherapy in this study consisted of 66 Gy given in 24 fractions of 2.75 Gy. This was combined with chemotherapy, given either sequentially as two neo-adjuvant courses of cisplatin and gemcitabin or concomitantly with daily cisplatin (6 mg/m^2^). For patients who did not participate in the EORTC study, standard treatment was offered which was equal to the concomitant treatment arm.

Radiotherapy as sole treatment was offered if combined radio-chemotherapy was medically contra-indicated or if patients refused this option. In this situation an alternative radiotherapy schedule was used sometimes (60 Gy in 20 fractions of 3 Gy) [5].

Treatment outcome for patients with locally advanced irresectable or inoperable NSCLC treated between1995 and 2004 is analysed here in a retrospective study.

## Methods

Between 1995 and 2004 131 patients with inoperable NSCLC were accepted for accelerated radiotherapy with curative intent.

All patients were staged by means of physical examination, haematological counts, renal and liver function tests, chest X-rays and contrast-enhanced CT-scan of the thorax and upper abdomen. Pathological diagnosis was obtained by bronchoscopy or cytological puncture.

Pathological confirmation of involvement of mediastinal nodes was obtained by cytological punction or mediastinoscopy.

Lung function tests and Carbon Monoxide Diffusion Capacity (DLCO) were part of the routine work-up.

Bone-scan and CT-scan or MRI of the brain was performed if metastases at these sites were suspected. 18 FDG PET was introduced as a routine staging procedure in our clinic in 2004. Therefore the vast majority of patients was not staged by means of a PET scan.

To be eligible for high-dose radiotherapy with curative intent patients had to fulfil the following criteria: inoperable or irresectable NSCLC, T1-4, N0-2, M0 [9], pathologically proven NSCLC or clinical and radiological suspicion for malignancy, and ECOG performance score 0–2. Patients with unexplained weight-loss of more than 5% in 3 months or 10% in 6 months were excluded.

If the forced expiratory volume at 1 second (FeV1) was less than 1 litre or DLCO < 50%, eligibility depended on the result of the radiotherapy planning Dose-Volume Histogram of the lung. Decision-making was based upon the mean lung dose according to the publication of Kwa et al [10].

Patients were also excluded if the length of the oesophagus receiving 66 Gy exceeded 12 cm according to EORTC-08912 [7].

To be eligible for chemotherapy patients should have adequate renal and cardiac function.

If renal and/or cardiac functions were suboptimal patients could be accepted for daily low dose cisplatin administration.

Standard treatment consisted of high-dose radiotherapy with concomitant daily low-dose cisplatin[7,8]. Some patients received sequential chemo-radiation. If chemotherapy was contra-indicated or refused, radiotherapy was offered as single modality.

Patients with a superior sulcus tumour were discussed with the thoracic surgeon after chemo-radiation. In selected cases a resection was performed.

### Radiotherapy

All patients were planned by means of a CT-scan in treatment position.

The Gross Tumour Volume (GTV) was defined as the primary tumour and pathological lymph nodes with a short axis > 10 mm on the CT-scan.

The Boost Planning Target Volume (BPTV) included the GTV with a margin of 12–15 mm in the lung, depending on the respiratory movements as seen under fluoroscopy and 10 mm in the mediastinum. The Elective Planning Target Volume (EPTV) encompassed the BPTV and the first lymph node drainage group not considered as pathological with a margin of 12 mm.

In general the EPTV was irradiated with two opposite anterior-posterior and posterior-anterior conformal fields (AP-PA). The dose administered was 40 Gy in 20 daily fractions of 2 Gy.

All fractions were given 5 times a week.

The BPTV was irradiated with conformal fields. The daily fraction dose to the BPTV was 2.75 Gy, given in 24 fractions to a total dose of 66 Gy. For the first 20 fractions a concomitant boost technique was used. The EPTV received 2 Gy per fraction and an extra dose of 0.75 Gy was given in the same session to the BPTV. The planned overall treatment time (OTT) varied between 32 and 34 days. The dose was defined according to the ICRU 50 report [11]. The fractionation schedule in case of radiotherapy only, consisted of 20 daily fractions of 3 Gy to a total dose of 60 Gy with an overall treatment time of 26 to28 days.

The maximal dose to the spinal cord was 50 Gy in fractions of 2 Gy or the equivalent dose.

The maximal length of the oesophagus irradiated to 40 Gy, and to 66 Gy, was 18 cm, and 12 cm, respectively.

All patients were treated with 10 MV photon beams.

### Chemotherapy

Two schedules of chemotherapy were used. Concurrent chemotherapy consisted of cisplatin (6 mg/m^2 ^intravenously) administered 1–2 hours before each fraction of radiotherapy. The planned total dose of cisplatin was 144 mg/m^2^. In the sequential schedule patients started with chemotherapy consisting of two courses of gemcitabin (1250 mg/m^2 ^on days 1 and 8 and cisplatin 75 mg/m^2 ^on day 2). The second course started on day 22. The radiotherapy was given 3 to 4 weeks after the last gemcitabin dose, usually on day 57.

### Toxicity

Late oesophageal toxicity and radiation pneumonitis were scored according to the RTOG/EORTC criteria [12].

### Statistical analysis

Local recurrence-free, distant recurrence-free, and overall survival were calculated from the last day of radiotherapy.

Local recurrence was defined as a situation in which clinical or radiological signs of tumour progression were observed within the radiation portals. The Kaplan-Meier method was used to estimate the probability of local recurrence-free, distant recurrence-free and overall survival[13]. The log-rank test was used to test differences between groups.

To evaluate association between prognostic factors and overall survival a Cox proportional hazard analysis was performed to obtain hazard ratio's (HR) and 95% confidence intervals (CI). The prognostic factors that were evaluated were: sex, age (continuous), ECOG performance score (0–1 vs. 2), pathology stage (I vs. II vs. III), radiotherapy dose (55–60 Gy vs. > 60 Gy) and chemotherapy.

The prognostic factors were removed step by step from the model using the Wald statistic to assess statistical significance. A P-value equal to or < 0.05 was considered statistically significant.

Three patients who did not finish radiotherapy and received a dose less than 55 Gy were left out of the multivariate analysis.

Statistical analysis was performed with Statistical Package for Social Sciences, version 12.0 (SPSS, Chicago, IL, USA).

## Results

A total of 131 patients was treated with high-dose radiotherapy with or without chemotherapy.

In 56 cases the radiotherapy was combined with concomitant cisplatin and in 26 cases with 2 courses of neo-adjuvant cisplatin and gemcitabin. In total 49 patients received radiotherapy without chemotherapy.

A summary of patient characteristics is presented in table 1. The mean age was 66 years (range 30–85). For patients receiving concomitant chemo-radiation, sequential chemo-radiation or radiation alone the mean age was 62, 61, and 71 years, respectively. Twenty patients have presented with weight loss > 5% for which a possible not tumour related cause was available. Only 4 patients had a PET-scan for staging.

**Table 1:** Patient characteristics of 131 patients treated with high dose accelerated radiotherapy with or without chemotherapy

		**Group**		
**Patient characteristics**	**Number of patients**	**I**	**II**	**III**
Total number	131	56	26	49
Male/female	89/42			
mean age (years)	66	62	61	71
Elderly (>70 years)	55	16	7	32
Performance (ECOG scale)				
0/1/2	28/76/27			
				
*Pathology*				
Adenocarcinoma	18			
Squamous cell carcinoma	39			
Large cell carcinoma	60			
Undifferentiated carcinoma	8			
No pathology	6			
				
*TNM stage*				
I/IIB	15/15	1/7	2/0	12/8
IIIA/IIIB	68/32	29/18	20/4	19/10
Unclassifiable	1	1		

The FeV1 value was known for 119 patients, with a mean of 77% (range 30–125%). The DLCO was available for 77 patients with a mean of 78% (range 30–121%).

### Treatment characteristics

A summary of treatment parameters is presented in table 2.

**Table 2:** Radiotherapy characteristics of 131 patients treated with high-dose accelerated radiotherapy with or without chemotherapy

**Radiotherapy characteristics**	
Dose BPTV (Gy)	number of patients
< 50	3
55	5
60	25
63	1
66	96
67	1
	
Overall Treatment Time (days)	
<26	3
26–31	35
32–34	59
35–39	29
43–57	5

One patient received a dose of 67.5 Gy for compensation of a split period.

For 25 patients the total dose to the BPTV was 60 Gy in fractions of 3 Gy. One patient received 63 Gy in fractions of 3 Gy for compensation of a split period.

For 5 patients a dose of 55 Gy in daily fractions of 2.75 Gy was administered. For one patient the mean lung dose was considered too high for a dose of 66 Gy, for another patient the length of the oesophagus receiving a daily fraction of 2.75 Gy was 14.3 cm which was judged too long for a total dose of 66 Gy. For 3 patients it was not clear why the maximal dose was reduced to 55 Gy. For three other patients the dose given was below 50 Gy; in two patients because of progressive distant disease and another died during the treatment period for unknown reasons.

Among 86 patients the Elective Planning Treatment Volume (EPTV) was irradiated to a dose of 40 Gy in fractions of 2 Gy.

The mean overall treatment time (OTT) was 33 days (range 21–57). In 29 patients the OTT ranged between 35–39 days. This was due to logistic reasons (holidays, start on Thursday) in 21 patients, in five patients the treatment was interrupted for acute side effects and in three patients the reason for interruption was not clear. In 5 patients the OTT was between 43 and 57 days, in two patients the treatment was interrupted because of oesophagitis, in three patients due to infection.

Two patients of group II showed progressive disease during induction chemotherapy. They received the radiotherapy as planned and were evaluated within this group.

### Survival

**Figure 1:**
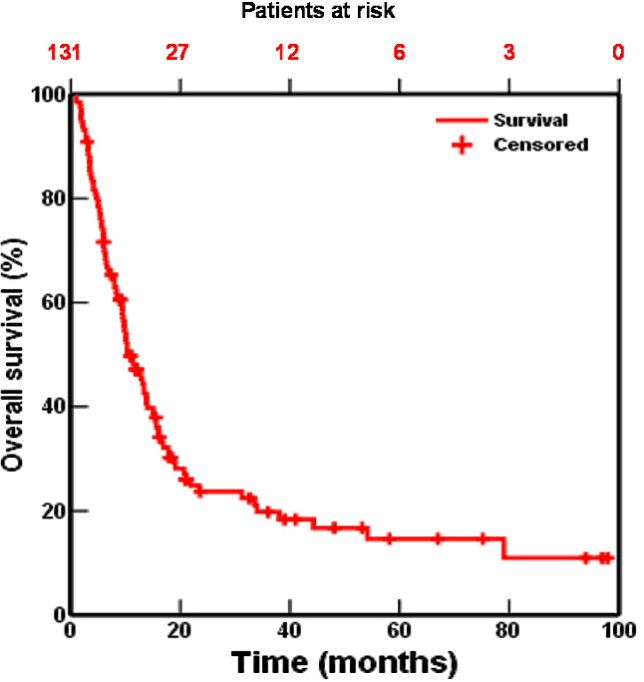
Actuarial overall survival of 131 patients treated with high dose accelerated radiotherapy with or without chemotherapy.

The median follow-up was 10.5 months. The 1, 2, and 5-year survival rates were 46%, 24%, and 15% respectively (fig 1). Disease-free survival at 1, 2, and 5 years was 38%, 30%, and 25%, respectively.

**Figure 2:**
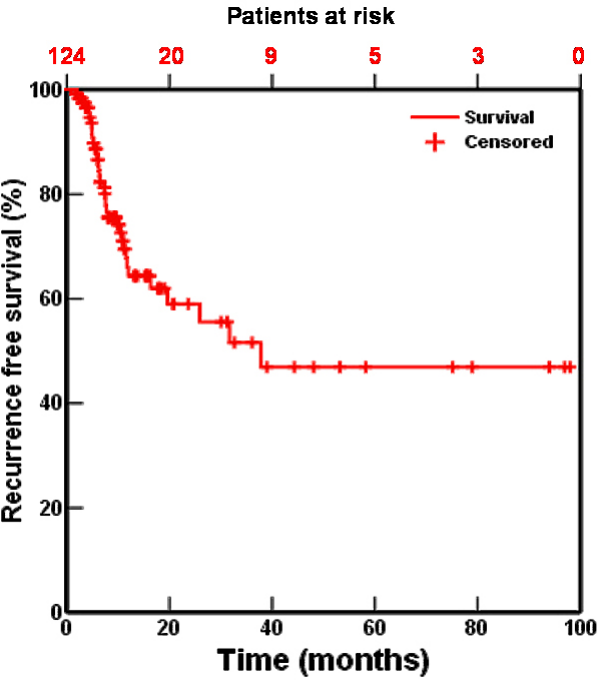
Local-recurrence free interval for 131 patients treated with accelerated high-dose radiotherapy with or without chemotherapy.

**Figure 3:**
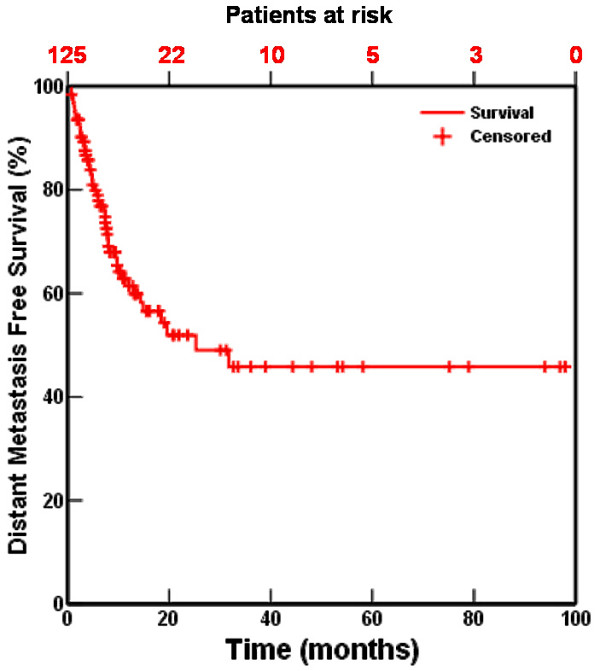
Distant-metastasis free interval for 131 patients treated with accelerated high-dose radiotherapy with or without chemotherapy.

The absolute incidence of a local recurrence was 36%, the incidence of distant metastases 46%. Local relapse-free interval at 1, 2, and 5 years was 64%, 56%, and 47%, respectively (fig 2). In 23 of the 131 patients no reliable data concerning a local relapse could be obtained. Distant relapse-free interval at 1, 2 and 5 years was 61%, 49% and 46%, respectively (fig 3). The number of remaining patients at 40 and 60 months is 10 and 5 respectively.

In 25 patients no reliable information about presence or absence of distant relapse was available. Twenty patients had a local as well as a distant relapse.

### Prognostic factors

**Figure 4:**
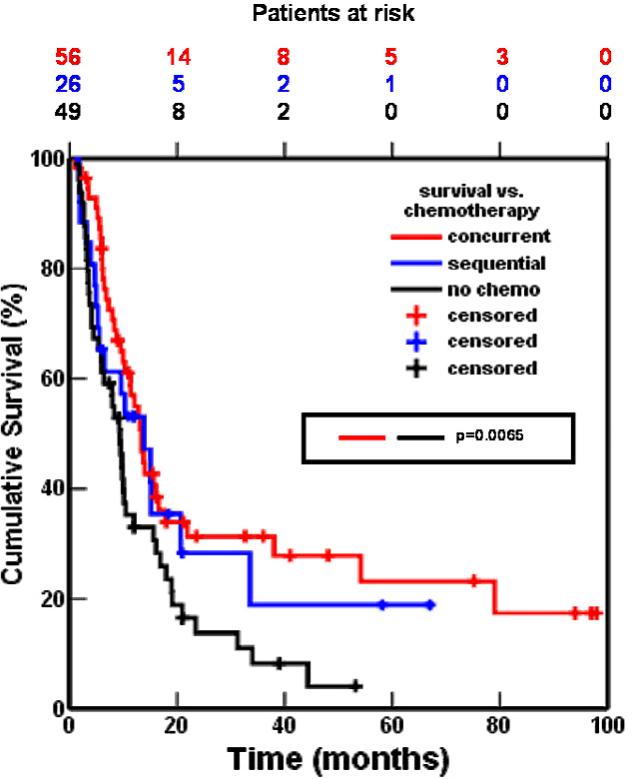
Actuarial overall survival of patients treated with high-dose accelerated radiotherapy with concomitant chemotherapy, with sequential chemotherapy or without chemotherapy.

Factors with a significantly favourable influence on overall survival were: chemo-radiotherapy (P = 0.01), performance status 0 or 1 (P = 0.04) and age < 58 years (P = 0.05). There was no statistically significant difference in OS between the two means of chemotherapy administration. When separately analysed only concomitant chemo-radiation yielded a significant survival benefit, compared to radiotherapy as single modality (median survival time 13.4 months (95% CI 10.7 to 16.1) vs. 9.4 months (95% CI 7.5 to 11.4); P = 0.01; figure 4). Patients receiving only radiotherapy were older and had more often an ECOG performance score more than 1.

At multivariate analysis only treatment with chemo-radiation was left as a prognostic factor for OS (P = 0.024). The HR for OS of sequential compared to concurrent chemotherapy was 1.18 (95% CI 0.65 to2.13; P = 0.583), and for no chemotherapy vs. concurrent chemotherapy 1.84 (95% CI 1.17 to2.88; P = 0.08). The radiation dose had no influence on the survival, irrespective of the treatment modality. The improved OS of chemo-radiation was apparent in patients up to 70 years as well as in patients > 70 years. Disease-free survival (DFS) at 5 years for patients who had received concomitant radio-chemotherapy was 30%. This was superior to the 5 years DFS for patients treated with sequential radio-chemotherapy (20%) and for the group treated with radiotherapy alone (18%), but the differences were not statistically significant. Age and stage had no influence on DFS.

For local relapse-free interval no influence of treatment modality could be seen. Distant metastasis-free interval was improved in patients treated with concurrent chemo-radiation, but the difference was not significant.,

At the moment of analysis 32 patients were alive. Fifteen patients had survived more than 36 months. Two underwent a lobectomy after the end of radiotherapy.

### Toxicity

For 19 patients data to assess late toxicity were not sufficiently available; for 13 patients the follow-up period was too short. Thus data about late toxicity are available for 99 patients (table 3).

**Table 3:** Late toxicity for 131 patients treated with high-dose accelerated radiotherapy with or without chemotherapy

**Late toxicity (RTOG/EORTC)**						
Grade	0	1–2	3	4	not evaluable	unknown
Number of patients	66	8	21*	4**	13	19
Group I	29	3	11	4	2	7
Group II	10	1	6	0	3	6
Group III	27	4	4	0	8	6

* pulmonary 17, oesophageal 3, cardiac 1

** pulmonary 1, oesophageal 2 (1 lethal), neuropathy 1

The four patients with late grade 4 toxicity were all treated with concurrent chemo-radiation. One patient died of uncontrolled complications of an oesophageal fistula. In this patient, with very extensive mediastinal nodal disease, the oesophagus was irradiated over a length of 14.3 cm to 66 Gy. Severe late toxicity was more frequent in patients treated with concurrent or sequential chemo-radiation (27% and 23% respectively) than in patients treated with radiotherapy only (8%).

Overall, severe late toxicity grade 3 did not occur more frequently in elderly patients. In the group of patients treated with concurrent chemo-radiation 9 out of 40 patients < 70 years presented severe late toxicity (23%); 5 of the 16 elderly patients in this group had severe late toxicity (31%). All patients presenting late toxicity grade 4 were younger than 70 years.

## Discussion

Our overall survival rates are in accordance with recently published results by other authors [8,14-22]. Survival in our series is significantly better among patients treated with a combination of chemo- and radiotherapy which is the only prognostic factor in multivariate analysis. An effect of selection bias cannot be ruled out, since concomitant chemo-radiation was standard treatment in our department. At the interpretation of the data we must be aware of the large number of censored patients after 40 months.

No significant difference in survival between concomitant and sequential administration of chemotherapy is observed in our series. The optimal way of combining chemotherapy with radiation is studied in several phase III studies and a meta-analysis is underway. These studies indicate that concurrent administration of chemotherapy and radiotherapy is superior to sequential administration [15-18,22-25]. The Locally Advanced Multi-Modality Protocol also demonstrated the superiority of concurrent CRT [26]. EORTC study 08972/22973 was underpowered to detect a significant difference between concurrent and sequential chemo-radiotherapy, but showed good results in both arms [8]. In the present study we were able to reproduce the results of the EORTC trial in a non-selected patient population outside of the setting of a randomised trial. In our series the 5-year overall survival is 23% for the concomitant CRT schedule, which is comparable to the 5-year survival data of concomitant chemo-radiotherapy of others [17,18,22]. Curran *et al. *do not report 5-year overall survival data, but a 2-year survival rate of 37% for patients treated concomitantly, which is somewhat higher than in our series [16]. All of these studies followed platinum-based multi-agent standard chemotherapy schedules with higher cumulative doses of cisplatin than in our study except for the EORTC trial.

There is no convincing evidence that concomitant standard poly-chemotherapy is superior to daily low-dose cisplatin alone, if it is combined with a high radiation dose. In one trial addition of concomitant cisplatin (daily 6 mg/m^2^) showed no improvement, but the administered radiation dose was only 45 Gy [27]. Carboplatin might be less effective as a radiosensitizer for NSCLC, as several low-dose single agent carboplatin studies were negative. A two-drug combination (carboplatin plus etoposide) was better than single agent only for carboplatin based trials [27-34].

Epidemiological studies show that with increasing age the percentage of people treated with chemotherapy and chemo-radiation decreases and that treatment is an independent prognostic factor while age does not play a role in stage III and IV NSCLC [21,35-37]. Aupérin describes an improved effect of chemo-radiation compared to radiation alone for patients older than 60 years with disease stage IIIB [27].

Can adjuvant chemotherapy improve survival? Recent trials and a meta-analysis have shown that adjuvant chemotherapy given post-operatively improves survival [38-40]. In contrast, neo-adjuvant chemotherapy given before concomitant chemo-radiation does not improve prognosis [26,41,42]. The data of Keene *et al*. also support the use of adjuvant chemotherapy after chemo-radiation with a daily low-dose of cisplatin [43].

Therefore adding adjuvant chemotherapy afterwards to our standard treatment of 66 Gy together with a daily low-dose of cisplatin might improve results.

Can accelerated radiotherapy improve results? The short OTT of 33 days and the high Biological Equivalent Dose (84 Gy for an α/β ratio of 10 Gy which is equivalent to a dose of 70 Gy in fractions of 2 Gy) might have been a favourable factor in our treatment outcome. In the CHART-study reduction of the OTT from 6 weeks to 12 days resulted in improved outcome with radiotherapy alone, indicating an influence of repopulation [44]. Several other studies report about improved results while shortening the OTT, for radiotherapy alone or for chemo-radiation [18,45-47]. Assuming that accelerated repopulation begins 28 days after start of treatment and that each day of prolongation hereafter should be compensated by 0.5 Gy, 66 Gy administered in 32 days could be equivalent to a BED_10 _of 93 Gy [Dische 02, Hermann 04, Fowler 04].

Dose-relationship for local control and survival has been clearly demonstrated for lung cancer [19]. In the Cochrane analysis the superior effect of chemo-radiation is independent of the radiation dose administered [24]. This suggests that increasing the radiation dose during concomitant treatment schedules might have a positive effect on local control, as was demonstrated by Socinsky [21].

Keene, Schild and Jeremic have reported 5-year survival of 20–25% for treatment schemes consisting of high-dose radiotherapy in a short OTT combined with daily administration of low-dose cisplatin (Keene, Schild) or concomitant poly-chemotherapy in a weekly schedule [36,43,48].

In our series some of the patients (non-PET-scan staged) had stage I-IIB, but stage did not show any association with survival among our patients treated with chemo-radiation.

Performance status and age are well known as prognostic factors for survival [21,35-37]. After correction for treatment modality, the significance of age and performance status disappears in our study.

Local recurrence-free and distant metastasis-free interval are in agreement with other reported series [49]. The presence of a local recurrence in a retrospective analysis is often difficult to determine. For local recurrence clinical or radiological signs of progression within the radiation portals had to be evident. A bronchoscopy to check presence or absence of local tumour was not performed as a routine procedure and only done in a minority of the patients. Besides, in a substantial number of patients reliable data about a local or distant relapse were missing. Therefore these figures have to be regarded with caution. The absence of information during follow-up in several patients and the difficulty in interpretation of the local situation in others can explain the lack of significant influence of treatment modality upon local relapse-free or distant relapse-free interval.

Results of radiotherapy as single modality are disappointing, with a median survival of 10 months and a 2 year actuarial survival of 15%. The administered radiation dose is relatively high however (BED10 78 Gy–84 Gy). Selection might partly explain this effect, while radiation mono-therapy was advised if chemo-radiation was not feasible. Higher doses are needed for improved survival. Pilot studies of dose escalation yielded promising data [19,50-53].

Results of patients presenting with ECOG performance score of 2 are poor, the median survival is 8 months and no one survived 2 years. We conclude that high dose radiotherapy with or without chemotherapy is not indicated for this group of patients.

### Toxicity

In our series the most severe late toxicity seems to be related to the oesophagus. This is in agreement with data in the literature of concomitant chemo-radiotherapy [24]. However, in EORTC study 08844 no increase in oesophageal toxicity was observed after daily or weekly administration of cisplatin [4]. The total dose of 55 Gy in this trial was much lower however. Keene *et al. *conclude that the addition of low-dose daily cisplatin 6 mg/m^2 ^to a radiotherapy dose of 69.6 Gy, given in fractions of 1.2 Gy twice daily, did not increase oesophageal toxicity [43]. On the contrary Belderbos *et al*. conclude that chemo-radiation of concomitant low-dose cisplatin increases the risk for acute oesophageal toxicity [54]. A relationship between acute and late oesophageal toxicity has been described by Singh *et al. *and Ahn *et al. *[55,56]. Other factors which correlate with the risk for oesophagitis are the absolute maximum radiation dose, volume of the oesophagus receiving 35 Gy ore more (V35) and the length of the irradiated oesophagus [54-57]. In our treatment protocol the length of the oesophagus receiving a dose of 66 Gy was restricted to 12 cm. In the patient with lethal late oesophageal toxicity the normal tolerance was wittingly exceeded.

According to the RTOG/EORTC criteria 17 patients developed a severe radiation pneumonitis grade 3 for which antibiotics and steroid treatment were given, but none of them developed a respiratory insufficiency; therefore we think that this toxicity is clinically acceptable. Decrease in toxicity is possible by a reduction of the planning target volume (PTV). If patients are staged with a PET-CT scan elective treatment can be omitted and a better definition of the GTV is possible. Both factors lead to a smaller PTV [58].

Severe late toxicity was not observed more frequently in elderly patients if treated with concurrent chemo-radiation. In our experience patients with suboptimal renal or cardiac function, as is often the case in elderly and frail patients, can still be eligible for administration of low-dose cisplatin, while standard poly-chemotherapy is not possible. Besides, the incidence of severe haematological toxicity of chemo-radiation with daily low dose cisplatin is low and this treatment can be given on an out-patient base [8,19]. Therefore we conclude that this schedule a good option for elderly people.

Furthermore the toxicity profile of this schedule makes it suitable for combination with biological response modifiers as for instance cetuximab, which has proven activity if combined with radiotherapy and which is tested in phase II and III trials in combination with chemo-radiation in Head and Neck cancer [59,60].

**In conclusion**: implementation of the treatment schedule used in EORTC study 08972/22973 in a non-randomised setting resulted in comparable outcome in our department. Treatment of patients with locally advanced NSCLC with concomitant chemo-radiation led to an actuarial 5-year survival of 23%. This was obtained with a radiation dose of 66 Gy, given in 24 fractions within a short overall treatment time and administration of single agent daily cisplatin 6 mg/m^2^. Toxicity of this treatment scheme is acceptable, if constraints are made for the volume of the irradiated oesophagus and the lungs. An important finding in this era of increasing age is that this treatment scheme is also feasible for elderly patients with a suboptimal renal and/or cardiac function. Thus, the preferred standard treatment of patients with locally advanced NSCLC in our department remained concomitant chemo-radiation with a low dose of daily cisplatin. Furtherways for improvement are escalation of the radiotherapy dose and/or reduction of the overall treatment time by acceleration and hyper-fractionation among patients staged by PET-CT, combination of accelerated and hyper-fractionated radiotherapy with concurrent chemotherapy, and adjuvant chemotherapy. Other new modalities which are under investigation are combinations of chemo-radiation with biological response modifiers.

## Competing interests

The author(s) declare that they have no competing interests.

## Authors' contributions

All authors have contributed substantially to conception and design, analysis and interpretation of data and to drafting and revising the article.

All authors read and approved the final manuscript.
